# Global DNA methylation is not elevated in blood samples from Machado-Joseph disease mutation carriers

**DOI:** 10.1080/15592294.2024.2368995

**Published:** 2024-06-20

**Authors:** Luís Teves, Ana Rosa Vieira Melo, Ana F. Ferreira, Mafalda Raposo, Carolina Lemos, Conceição Bettencourt, Manuela Lima

**Affiliations:** a Faculdade de Ciências e Tecnologia, Universidade dos Açores, Ponta Delgada, Portugal; b Instituto Ciências Biomédicas Abel Salazar (ICBAS), Universidade do Porto, Porto, Portugal; c UnIGENe, Instituto de Investigação e Inovação em Saúde (i3S), Porto, Portugal; d Department of Neurodegenerative Disease and Queen Square Brain Bank for Neurological Disorders, UCL Queen Square Institute of Neurology, London, UK

**Keywords:** Machado-Joseph disease, SCA3, polyq disorders, DNA methylation, epigenetic modifiers

## Abstract

Machado-Joseph disease (MJD) is an autosomal dominant spinocerebellar ataxia (SCA) caused by a polyglutamine expansion in the ataxin-3 protein, which initiates a cascade of pathogenic events, including transcriptional dysregulation. Genotype-phenotype correlations in MJD are incomplete, suggesting an influence of additional factors, such as epigenetic modifications, underlying the MJD pathogenesis. DNA methylation is known to impact the pathophysiology of neurodegenerative disorders through gene expression regulation and increased methylation has been reported for other SCAs. In this work we aimed to analyse global methylation in MJD carriers. Global 5-mC levels were quantified in blood samples of 33 MJD mutation carriers (patients and preclinical subjects) and 33 healthy controls, matched by age, sex, and smoking status. For a subset of 16 MJD subjects, a pilot follow-up analysis with two time points was also conducted. No differences were found in median global 5-mC levels between MJD mutation carriers and controls and no correlations between methylation levels and clinical or genetic variables were detected. Also, no alterations in global 5-mC levels were observed over time. Our findings do not support an increase in global blood methylation levels associated with MJD.

## Introduction

DNA methylation, mainly consisting of a methyl group addition to the cytosine carbon 5 of a CpG site, resulting in 5-methylcytosine (5-mC), is the most well studied epigenetic modification and is known to be involved in gene expression and alternative splicing regulation [1]. DNA methylation patterns are widely recognized as modulators of clinical and pathological features of several neurodegenerative disorders [2–4], including the currently untreatable inherited polyglutamine (polyQ) disorders, caused by exonic CAG expansions in the respective causative genes [5]. Reversibility of DNA methylation is opening new perspectives for the development of epigenetic therapies [6]; noteworthy, the use of DNA methylation inhibitors has been demonstrated to mitigate polyQ-induced neurodegeneration in mouse models of disease [7]. Studies investigating DNA methylation changes in the brain tissue of patients with neurodegenerative disorders have been limited by the scarcity of good quality *post-mortem* brain tissue and are prone to be biased towards the end-stage of the disease. Noteworthy, despite methylation displaying tissue specificity [8], overlapping changes in brain and blood have been described, namely in Huntington’s disease (HD) [9], prompting the investigation of peripheral methylation alterations in other polyQ disorders. Studies of global methylation, i.e., the total 5-mC content in a sample relative to total cytosine content, have evidenced elevated levels in other polyQ diseases, namely spinocerebellar ataxias type 1 and 2 (SCA1 and SCA2, respectively) [10]. DNA methylation studies, however, are scarce [11–15] in Machado-Joseph disease (MJD)/spinocerebellar ataxia type 3 (SCA3) carriers. MJD, the second most frequent polyQ disease, after HD [16], is a late-onset autosomal dominant neurodegenerative polyQ ataxia, caused by the expansion of a CAG repeat tract in exon 10 of the *ATXN3* gene [17]. The ubiquitously expressed mutant ataxin-3 interferes with cellular homoeostasis and initiates a cascade of pathogenic events [18], including transcriptional dysregulation, which has been detected in blood samples from MJD mutation carriers [19–23], at very early disease stages and even prior to disease onset [23]. Currently untreatable, MJD displays marked clinical heterogeneity, namely a wide variation of age at onset (AO), which is only partially correlated (~50% to ~ 70%) with the size of the CAG tract [24]. Although several genetic modifiers have been already identified (reviewed in [25], they only add a small fraction to the variance in the age at onset explained by the CAG tract [25]. The extent to which putative differences in DNA methylation between MJD carriers (from preclinical – in which ataxia is absent – to clinically manifest disease), yet to be studied, could predict MJD status or contribute to transition for clinically manifest disease remains unknown. Here we perform a study of the global 5-mC levels in blood samples from carriers of the MJD mutation, including preclinical subjects and patients, compared to matched controls to gain insights into methylation changes associated with MJD.

## Subjects and methods

### Subjects

Blood samples from a total of 33 carriers of the MJD mutation (14 preclinical subjects and 19 patients) of Azorean origin and living in the Azores were used in this study. Preclinical subjects are mutation carriers with no reported complaints and/or a SARA score (Scale for the Assessment and Rating of Ataxia) <3 [26]. Preclinical subjects were molecularly confirmed as carriers of the *ATXN3* mutation, in the context of the Azorean Genetic Counselling and Predictive Test Program (Regional Health System). The number of CAG repeats at the *ATXN3* gene was determined according to Bettencourt and colleagues [27]. Although that are other relevant components of MJD clinical heterogeneity (e.g., parkinsonism), age at onset, disease duration, CAG tract length and SARA score were selected to be analysed, since these variables are the most widely documented. For patients, the age at onset was defined as the age of first appearance of ataxia, reported by the patient or a by a caregiver. SARA score values were available for 10 patients. For a subset of 16 MJD subjects, samples from two collection points, baseline, and follow-up visit (with a range of 1 to 5 years between collection points) were available for a follow-up analysis. To account for environmental context, blood samples from 33 healthy controls of Azorean origin and living in the Azores were matched by age (age at collection), sex, and smoking status (smoker, former smoker, and never smoked) with the MJD carriers. A summarized clinical, genetic, and demographic characterization of the participants is provided in Table 1.

**Table 1.** Characterization of the participants (controls, preclinical subjects, and patients) used in this study.

	Controls	Preclinical subjects	Patients
**Cross-sectional study (*n*=66)**			
n (Female; Male)	33^Ɨ^ (18; 15)	14 (8; 6)	19 (10; 9)
Age^1^, years	38.8 ± 13.6 [18; 71]	30.0 ± 6.2 [21; 43]	45.9 ± 14.0 [17; 73]
Smoking status^2^ (n)	S (14); F (3); N (16)	S (5); F (1); N (8)	S (9); F (2); N (8)
CAGn expanded allele^3^	NA	68.2 ± 2.8 [64; 75]	69.3 ± 3.9 [62; 78]
Age at onset, years	NA	NA	37.6 ± 12.3 [16; 60]
Disease duration, years	NA	NA	8.6 ± 5.4 [1; 20]
SARA score	NA	0.7 ± 0.8 [0; 2]*	12.3 ± 7.9 [3; 28.5]*
**Follow-up study (*n*=16)**			
n (Female; Male)	NA	6 (5;1)	10 (5;5)
Age baseline^4^, years	NA	35.0 ± 7.6 [21; 43]	39.1 ± 13.2 [17; 66]
Age visit 1^5^, years	NA	37.4 ± 6.8 [28; 47]	41.1 ± 14.5 [19; 71]
Smoking status^2^ (n)	NA	S (2); F (0); N (4)	S (6); F (1); N (3)
CAGn expanded allele^3^	NA	68.8 ± 3.3 [66; 75]	71.2 ± 3.3 [67; 78]
Age at onset, years	NA	NA	32.1 ± 10.3 [16; 50]
Disease duration 1^6^, years	NA	NA	7.0 ± 6.3 [1;20]
Disease duration 2^7^, years	NA	NA	10.0 ± 7.4 [1;23]

Continuous variables are shown as mean ± standard deviation [minimum; maximum]; ^1^Age at blood collection; ^2^Smoking status in three categories: S (Smoker), F (Former smoker), N (Never smoked); ^3^Number of CAG repeats in expanded allele of MJD carriers; ^4^Age at first blood collection; ^5^Age at second blood collection; ^6^Disease duration at baseline in follow-up study; ^7^Disease duration at visit 1 in follow-up study; ^Ɨ^Age (±3 years) and sex-matched paired controls for preclinical subjects and patients; *Information available for 10 patients and eight preclinical subjects; SARA = Scale for the assessment and rating of ataxia; NA, not applicable/not available.

### Methods

Global DNA methylation levels (global 5-mC) were quantified with the 5-mC DNA ELISA Kit (Zymo Research), according to manufacturer’s specifications. The samples were assayed in duplicate using 200 ng of gDNA per sample, including the standards (for standard curve). The absorbance values were measured using a ThermoFisher microplate reader at a wavelength of 405 nm, 40 minutes after adding the horseradish peroxidase (HRP) developer. A standard curve was generated for each assay to determine the 5-mC percentage and global methylation was calculated following the manufacturer’ instructions. Comparisons of median global 5-mC levels between overall MJD mutation carriers and controls, as well as between patients and preclinical subjects and their respective controls, were performed using a Mann-Witney test (two-tailed). Generalized linear models adjusting for age were performed to assess differences in global 5-mC levels between patients and preclinical subjects, and patients with short (<8 years, *n* = 9) and long disease duration (≥8 years, n = 10); the median disease duration among patients was 8 years (IQR: 4–11 years). Spearman’s correlations (two-tailed) were carried out between 5-mC levels and: a) the age at onset (adjusted for age and GAG expansion); and b) the SARA score (adjusted for age and disease duration, n = 10). Spearman’s correlations (two-tailed) were also conducted between 5-mC levels and age in the MJD carrier’s group and the total control group. The comparison of 5-mC levels between the two sampling moments was performed using a linear mixed model [28], adjusted for age, sex and smoking status. Statistical analyses were performed in IBM SPSS Statistics for Windows, version 29. The study graphs were performed in the GraphPad Prism version 9.5.1 for Windows. Statistical significance was set at p < 0.05.

## Results

**Figure 1.**
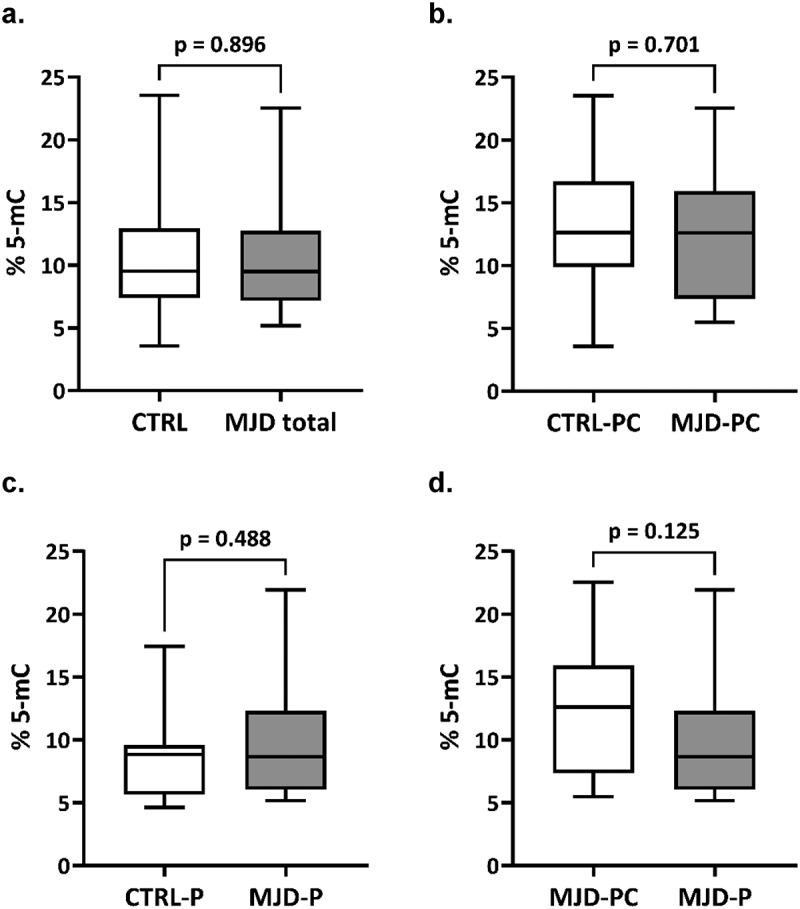
Global 5-mC levels in MJD mutation carriers. (a) Box plots for the global 5-mC levels in 33 MJD mutation carriers (MJD) and age-, sex- and smoking status-matched healthy controls (CTRL). (b) Box plots for the global levels of 5-mC in 14 preclinical subjects (MJD-PC) and age-, sex- and smoking status-matched healthy controls (CTRL-PC). (c) Box plots for the global 5-mC in 19 MJD patients (MJD-P) and age-, sex- and smoking status-matched healthy controls (CTRL-P). (d) Box plots for the global levels of 5-mC in 14 preclinical subjects (MJD-PC) and 19 MJD patients (MJD-P).

**Figure 2.**
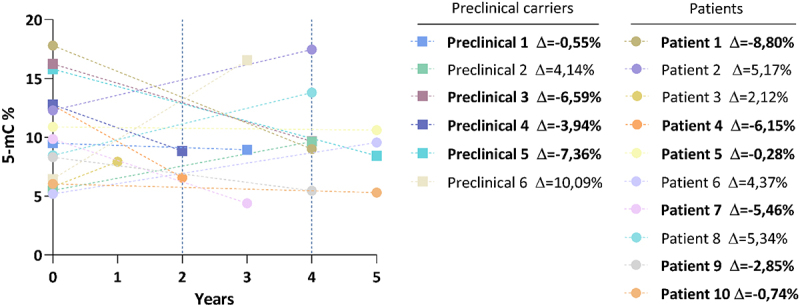
Follow-up analysis of global 5-mC levels in MJD mutation carriers. 5-mC levels in the two collection timepoints (baseline and visit 1) for each MJD subject (n=16), including patients 6 preclinical carriers and 10 patients. The delta value is the difference of the global 5-mC % between visit 1 and the baseline for each MJD subject. MJD subjects with a decrease in global 5-mC levels are highlighted in bold.

Levels of global DNA methylation (global 5-mC) of total MJD mutation carriers (n = 33) were similar to those of their pair-matched controls. The median methylation levels (95% confidence intervals) were 9.50 [9.16–12.6] for MJD mutation carriers and 9.53 [8.88, 12.2] for controls (Figure 1(a)). Global 5-mC were also similar when comparing the preclinical group (n = 14) and their controls (Figure 1(b); 12.6 [9.24, 15.2] and 12.6 [9.98, 16.2], respectively) or when considering the MJD patients (n = 19) with their controls (Figure 1(c)); 8.66 [7.74, 12.0] and 8.84 [7.18, 10.2], respectively). Global methylation levels between preclinical subjects and patients were similar after adjusting for age. Methylation levels were significantly inversely correlated with age in the total control group rho = −0.349, p = 0.047), but not in the group of MJD carriers rho = −0.315. p = 0.074). In patients, global 5-mC levels were not associated with clinical variables (disease duration, age at onset, CAG tract, or SARA score; data not shown). The follow-up analysis of the 16 MJD subjects showed that methylation levels at baseline ranged from 5.2% to 17.8%. Although a decrease in the global 5-mC levels was observed in 10 of the 16 MJD subjects (6 out of the 10 patients and 4 out of the 6 preclinical subjects), no longitudinal differences were found in global methylation levels when adjusting for the interval between baseline and visit 1 (p = 0.939) (Figure 2).

## Discussion/Conclusions

In this study, we investigated the existence of alterations in global methylation in blood samples from MJD mutation carriers. Our study shows no evidence of an elevation in global methylation levels in MJD, contrarily to the previously reported for other polyQ ataxias [10]. Moreover, no differences in global methylation levels were detected between preclinical subjects and patients, after adjusting for age. In addition, we could not detect an association between methylation levels and clinical variables, namely disease duration, age at onset, CAG tract or SARA score. A previous study in SCA1 and SCA2 reported significantly higher global 5-mC levels in blood samples from patients of both diseases, using a smaller sample size (SCA1 n = 17 patients; SCA2 n = 28 patients) and employing an ELISA methodology with comparable performance to that used in our study [10]. Nevertheless, in line with our results, in a set of 21 Huntington disease patients and 81 controls, no changes in global methylation levels were detected [10]. This seemingly heterogeneous behaviour -an increase in global methylation is observed in some polyQ disorders, but not in others – points to putative differences in the role of methylation in this group of disorders. Several molecular alterations have been detected in the preclinical phase of MJD [23]; therefore, although no global methylation changes were detected in this stage, a more in-depth analysis should include a larger number of preclinical subjects. In fact, detecting alterations in the earliest stages of MJD may help elucidating the involvement of DNA methylation in disease pathogenesis and clinical phenotype. Global quantification of DNA methylation by ELISA is useful for screening samples for changes in DNA methylation related to disease, and is cost-effective and easy-to-use, providing a rough evaluation of DNA methylation [29]; however, it present some limitations: a) only detects methylation at a global level but cannot identify specific CpG site methylation changes. Thus, a potential dysregulation of 5-mC patterns may go undetected if it involves both hypermethylation and hypomethylation in different genomic regions, nullifying their overall impact; and b) detects a significant amount of mitochondrial DNA methylation and overestimate the global genomic DNA methylation levels [30]. Global 5-mC analyses can be biased due to the cell type composition of a heterogeneous tissue such as blood, as different blood cell types have significantly different numbers of mtDNA copies [31]. Therefore, this can contribute to masking or amplifying the 5-mC levels under analysis, making it more challenging to clarify the potential association with a specific disease. Results from this study cannot rule out the possibility of global DNA methylation changes associated with MJD, as these might be detected in brain tissue, the area affected by the disease. Putative DNA methylation differences in MJD blood samples may be revealed through a more in-depth methodology, such as microarray-based DNA methylation profiling techniques, which enable the detection of methylation changes at a gene position resolution [32]. Moreover, investigating other epigenetic changes in addition to local DNA methylation is mandatory. Our study included an exploratory analysis of a subset of MJD subjects for which follow-up data was available. We found no significant variation of global methylation from baseline to visit 1; noteworthy, the trend for a decrease in global methylation levels from baseline to follow-up visit may be related with age. Indeed, a study that analysed global methylation variation related to age in human blood cells reported age-associated hypomethylation [33]. In fact, we found an inverse correlation of global methylation levels with age in the control group. The absence of such correlation in the MJD group may be due to small elevations in global 5-mC levels, which led to the loss of this correlation. However, these changes are not pronounced enough to be considered distinct in blood samples of MJD carriers. Future follow-up studies should include controls, a larger overall sample size, and more evaluation time points to enhance our understanding of global methylation patterns during disease progression, challenging goals for a rare disease. In summary, no global methylation changes associated with MJD were found in blood samples. Although we cannot exclude the presence of methylation changes associated with MJD, these seem to be of lower magnitude than those previously observed in other polyQ ataxias.

## Supplementary Material

Supplementary material.pdf

## Data Availability

The data presented in this study are available on request to the corresponding author.
