# Supplementary material for: Global DNA methylation is not elevated in blood samples from Machado-Joseph disease mutation carriers
Source: Epigenetics. 2024 Jun 20;19(1):2368995. doi: 10.1080/15592294.2024.2368995 (PMC11195492; doi:10.1080/15592294.2024.2368995)
Supplement: Supplementary material.pdf [file KEPI_A_2368995_SM6100.pdf]

Table A1. Characterization of the participants (controls, preclinical subjects, and patients) used in this study.

	**Controls**	**Preclinical subjects**	**Patients**
**Cross-sectional study (n=66)**			
n (Female; Male)	33[†] (18; 15)	14 (8; 6)	19 (10; 9)
Age[1], years	38.8 ± 13.6 [18; 71]	30.0 ± 6.2 [21; 43]	45.9 ± 14.0 [17; 73]
Smoking status[2] (n)	S (14) ; F (3) ; N (16)	S (5) ; F (1) ; N (8)	S (9) ; F (2) ; N (8)
CAGn expanded allele[3]	NA	68.2 ± 2.8 [64; 75]	69.3 ± 3.9 [62; 78]
Age at onset, years	NA	NA	37.6 ± 12.3 [16; 60]
Disease duration, years	NA	NA	8.6 ± 5.4 [1; 20]
SARA score	NA	NA	12.3 ± 7.9 [3; 28.5]*
**Follow-up study (n=16)**			
n (Female; Male)	NA	6 (5;1)	10 (5;5)
Age baseline[4], years	NA	35.0 ± 7.6 [21; 43]	39.1 ± 13.2 [17; 66]
Age visit 1[5], years	NA	37.4 ± 6.8 [28; 47]	41.1 ± 14.5 [19; 71]
Smoking status[2] (n)	NA	S (2) ; F (0) ; N (4)	S (6) ; F (1) ; N (3)
CAGn expanded allele[3]	NA	68.8 ± 3.3 [66; 75]	71.2 ± 3.3 [67; 78]
Age at onset, years	NA	NA	32.1 ± 10.3 [16; 50]
Disease duration 1[6], years	NA	NA	7.0 ± 6.3 [1;20]
Disease duration 2[7], years	NA	NA	10.0 ± 7.4 [1;23]

Continuous variables are shown as mean ± standard deviation [minimum; maximum]; [1]Age at blood collection; [2]Smoking status in three categories: S (Smoker), F (Former smoker), N (Never smoked); [3]Number of CAG repeats in expanded allele of MJD carriers; [4]Age at first blood collection; [5]Age at second blood collection; [6]Disease duration at baseline in follow-up study; [7]Disease duration at visit 1 in follow-up study; [†]Age (±3 years) and sex-matched paired controls for preclinical subjects and patients; *Information available for ten patients; SARA = Scale for the assessment and rating of ataxia; NA, not applicable/not available.

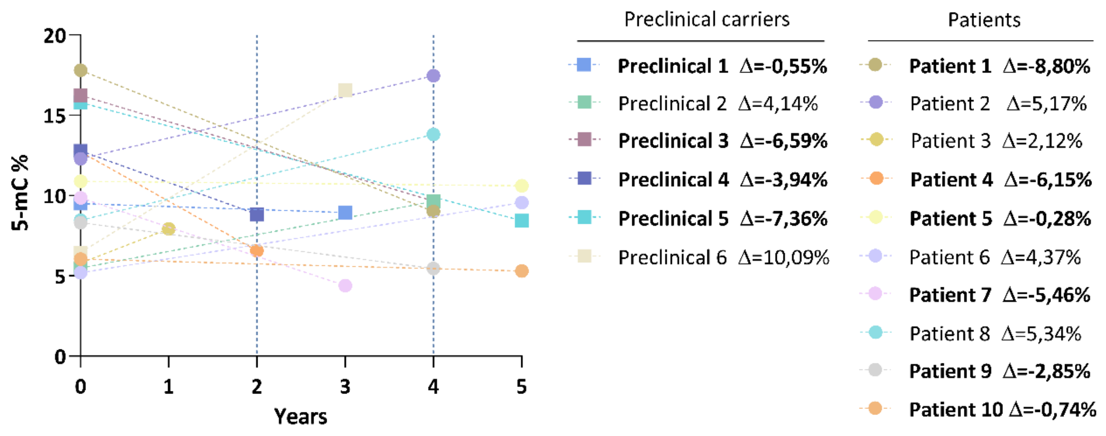

Figure A1. Follow-up analysis of global 5-mC levels in MJD mutation carriers. 5-mC levels in the two collection timepoints (baseline and visit 1) for each MJD subject (n=16), including patients 6 preclinical carriers and 10 patients. The delta value is the difference of the global 5-mC % between the baseline and visit 1 for each MJD subject. MJD subjects with a decrease in global 5-mC levels are highlighted in bold.
